# The Impact of Reduced Fire Risk Cigarettes Regulation on Residential Fire Incidents, Mortality and Health Service Utilisation in New South Wales, Australia

**DOI:** 10.3390/ijerph191912481

**Published:** 2022-09-30

**Authors:** Nargess Ghassempour, Wadad Kathy Tannous, Kingsley Emwinyore Agho, Gulay Avsar, Lara Ann Harvey

**Affiliations:** 1 School of Business, Western Sydney University, Parramatta, NSW 2150, Australia; 2 Rozetta Institute Group, The Rocks, NSW 2000, Australia; 3 Translational Health Research Institute, Western Sydney University, Campbelltown, NSW 2560, Australia; 4 School of Health Sciences, Western Sydney University, Penrith, NSW 2751, Australia; 5 Fall, Balance and Injury Research Centre, Neuroscience Research Australia, Randwick, NSW 2031, Australia; 6 School of Population Health, University of New South Wales, Kensington, NSW 2033, Australia

**Keywords:** residential fires, cigarette fires, smokers’ materials fires, reduced fire risk cigarettes regulation

## Abstract

Smoking materials are a common ignition source for residential fires. In Australia, reduced fire risk (RFR) cigarettes regulation was implemented in 2010. However, the impact of this regulation on residential fires is unknown. This paper examines the impact of the RFR cigarettes regulation on the severity and health outcomes of fire incidents in New South Wales (NSW), Australia, from 2005 to 2014. Fire department data from 2005 to 2014 were linked with ambulance, emergency department, hospital, outpatient burns clinic and mortality datasets for NSW. Negative binomial regression analysis was performed to assess the changes to fire incidents’ severity pre- and post-RFR cigarettes regulation. There was an 8% reduction in total fire incidents caused by smokers’ materials post-RFR cigarettes regulation. Smokers’ materials fire incidents that damaged both contents and structure of the building, where fire flames extended beyond the room of fire origin, with over AUD 1000 monetary damage loss, decreased by 18, 22 and 12%, respectively. RFR cigarettes regulation as a fire risk mitigation has positively impacted the residential fire incident outcomes. This provides support for regulation of fire risk protective measures and bestows some direction for other fire safety policies and regulations.

## 1. Introduction

Globally, fires are a common cause of emotional distress, physical injury and death, and studies show that residential fires are a major cause of fire-related morbidity and mortality [1]. One of the main risk factors for residential fires is dropped or improperly discarded cigarettes [2]. These types of fires are usually caused by smokers falling asleep with a lit cigarette or dropping their cigarettes or discarding them negligently [3,4,5,6].

In 2012, in the United States, according to the US Fire Administration, 14% of all fire deaths were in residential buildings and 2% of all residential fires were due to cigarette smoking [2]. In Australia, annually, more than 4500 fires are caused by cigarettes and between 2000 and 2005, 77 people died in fires that were started by cigarettes [7]. In another study, it was shown that from a total number of 900 residential fire-related deaths during 15 years from 2003 to 2017 in Australia, 161 (18%) deaths were caused by smokers’ materials such as cigarettes and pipes [8]. 

Using National Coronial Information System (NCIS) data, Hoy and Morton (2006) showed that unintentional cigarette-related fires averaged 11 deaths each year in Australia. More recently, for the period 2003 to 2017, Coates et al. (2019) used the same data and determined that one-quarter of fires in Australia had been caused by smoking material (i.e., cigarettes, pipes), with over a third of those relating to smoking in bed [8]. 

**Figure 1:**
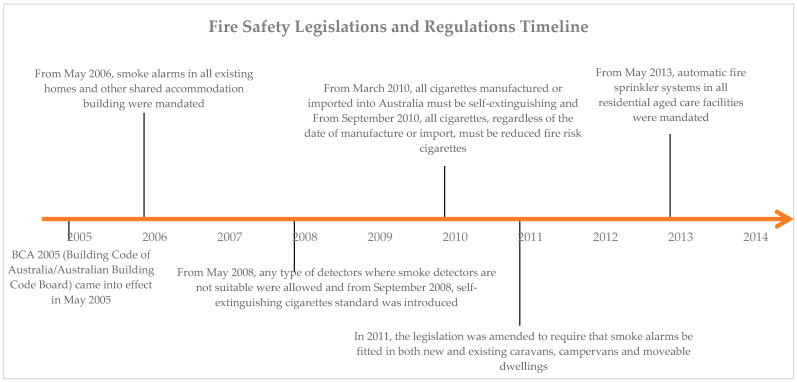
Fire safety legislation and regulations, and changes in Australian building codes in NSW from 2005 to 2015.

In the past decade, in many countries including Australia, reduced fire risk (RFR) cigarettes also known as fire safe cigarettes (FSC), lower ignition propensity (LIP) and reduced ignition propensity standard designs, were introduced to reduce the risk of smoking-related fires [9,10,11]. In 2010, Australia mandated that all cigarettes sold and imported meet the RFR regulation of 2008 and be self-extinguished [see Figure 1]. This was as a result of a number of studies conducted by Hoy and Marten (2006) [12]. They showed that fires caused by cigarettes take 11 lives on average each year in Australia. These findings helped lead to an endorsement by state and federal emergency services ministers for a national mandatory standard for the manufacture of reduced fire risk cigarettes This standard has been legislated by government and came into effect in March 2010 [13].

In 2012, Fire and Rescue NSW found that in FRNSW areas, the average number of cigarette-related fires reduced from 121 to 57 each month since the introduction of reduced fire risk cigarettes. They also found that there has been a dramatic 53% drop in the number of cigarette-related fires from 1490 in 1988 to 716 in 2011 [14]. They attributed the reduction in cigarette-related fires to fewer smokers, upholstery fabric regulations and mandatory smoke alarms, suggesting it is too early to judge if RFR cigarettes are contributing to this significant decline. Some researchers have studied the RFR cigarettes regulation [2,7,10,11,15,16,17] but to date, few studies have explored the impact of this regulation in terms of fire incidents and their health outcomes [10,11,18]. In Australia and NSW specifically, research has not been conducted on the impact of RFR cigarettes regulation on residential fire incidents and resulting injuries and deaths. 

A limited number of Australian studies that examined the impact of other legislation and regulations on residential fire incidents includes a Harvey et al. (2013) ecological-level study that examined the impact of smoke alarm take-up and observed that an increase in smoke alarm ownership coincided with a decrease in injuries as measured by the number of residential fire-related presentations to the hospital [19,20]. The study used a combination of survey data from the NSW Population Health Survey, weighted to represent the NSW population, and hospital admission data. However, as the two datasets were not linked, survey respondents may not necessarily be the ones that presented to the hospital. 

This study aims to assess the impact of the introduction of RFR cigarettes regulation on the number, intensity and severity of residential fire incidents as well as the impact on health service use and deaths, by using fire department data linked with administrative health data for NSW, Australia. Findings from this study will enable public health researchers and Fire and Rescue NSW to understand the health impact of residential fire incidents caused by smokers’ materials further and to target and educate households with at least one smoker resident, to reduce smoking-related materials’ residential fires and their health impact further.

## 2. Materials and Methods

### 2.1. Study Population and Data Sources

New South Wales (NSW) is the most populous state in Australia, with a population of over 8.1 million people, of which more than 11% are smokers [21,22]. This study is a population-based cohort analysis of linked administrative data. This study used RESFIRES, a linked data asset comprising nine datasets of residential fire incidents in NSW for the period 1 January 2005 to 31 December 2014, details of which have been described elsewhere [23]. 

RESFIRES includes Fire and Rescue New South Wales (FRNSW) Australian Incident Reporting Systems (AIRS), Ambulance NSW, Emergency Department Data Collection (EDDC), NSW Admitted Patient Data Collection (APDC), NSW Statewide Burn Injury System (SBIS) and NSW Registry of Births, Deaths and Marriages (RBDM). Each is detailed below.

FRNSW AIRS contains information about residential fire incidents that were attended by the FRNSW brigade in major cities, metropolitan areas and towns. In that dataset, a residential fire incident is defined as a fire incident that occurred in a residential building and has been reported and to which a rescue team was dispatched. The specific variables that were used in this study include incident ID, ignition factor, form of heat of ignition, area of fire origin, extent of flame damage, type of incident, estimated percentage of property involved when arrived, estimated dollar loss and total number of brigade personnel at scene, alarm time and duties completed time [24]. Incident ID indicates the incidents that had individual(s) involved in that fire incident as AIRS records are per fire incident with each incident given a unique incident ID. Form of the heat of ignition is the form of heat energy which caused the ignition, which includes fuelled-fire, electrical equipment, smokers’ materials, open flame, hot object or friction, explosive/fireworks, natural source, spreading from another hostile, other forms of the heat of ignition and form of heat of ignition undetermined. The form of the heat of ignition as smokers’ materials excludes matches and lighters and has the sub codes (categories) of cigarette, cigar, pipe and heat from smokers’ materials not classified. In this study, we included fires that were caused by cigarette using the form of heat of ignition variable and smokers’ materials division. Ignition factor is defined by the AIRS data dictionary as the circumstances that permitted the heat source and combustible material to combine to start the fire and is based on the fire brigade personnel making a reasonable judgement of fire behaviour and cause [24]. This variable contains misuse of the heat of ignition division, which contains the following categories: abandoned, discarded material (included are discarded cigarettes and cigars) and falling asleep. In this study, we used records of incidents that were caused by abandoned, discarded materials where the form of heat from smokers’ materials was not classified.

The area of fire origin is defined as the area within the property where the fire originated. The extent of flame damage is defined as the extent of the area burned or charred by flame impingement and can be used to measure the magnitude of the fire, and is coded in increasing levels from 1—confined to the object of origin to 7—extended beyond the structure of origin. 

The type of incident is defined as that determined by the reporting authority after arriving at the scene, which includes structure only, contents only, and structure and contents.

Health data includes ambulance, hospitals, burns clinics and mortality data, and they were used to determine health service utilisation and deaths in the cohort. 

Ambulance data contains operational information from the Computer-Aided Dispatch (CAD) system, data documented by clinicians in the paper-based Patient Health Care Record (PHCR) and clinical and treatment information in the electronic medical record (eMR). EDDC contains records showing a presentation to an emergency department (ED) in NSW. 

Records of admission to hospitals were obtained from NSW APDC, which provides a census of hospital admissions to all NSW public and private hospitals. Data are collected on all episodes of care in the hospital which end with the discharge, transfer or death of the patient or when the service category for the patient changes (i.e., a change from acute care to rehabilitation for a patient during a stay in a single facility). Thus, for a single injury, an individual may have multiple episodes of care recorded. Records of outpatient burns clinics were obtained from the Statewide Burn Injury Service (SBIS) dataset. 

Mortality data contain records of all the deaths in NSW obtained from the RBDM and the Australian Bureau of Statistics (ABS) Cause of Death Unit Record File (COD-URF), which contains records of all deaths of NSW residents, and provides the date of death.

The data were linked by the Centre for Health Record Linkage (CHeReL) where individuals are identified with a unique project person number (PPN), details of which have been described elsewhere [23]. Ethics approval for this study was obtained from the NSW Population and Health Services Research Ethics Committee (HREC/16/CIPHS/36) and Western Sydney University Human Research Ethics Committee (RH12399).

In this study, we measured residential fire incidents due to abandoned cigarettes, fire incidents ignited due to falling asleep as well as in sleeping areas. The severity of the fire incident was tested based on the number of fire personnel who attended the fire incidents, the time they spent to attend fire, the percentage of the property involved when the fire personnel arrived, the type of fire incident damaging structure and contents of the property, the flame damage extending beyond the room of fire origin and the monetary damage loss due to fire flames and firefighting. The severity of fire was also measured based on the health impact in terms of health service utilisation, hospitalisation, length of stay (LOS) in hospital and deaths.

For the variables that were not categorical and there were no categories to distinguish severe and non-severe residential fire incidents, we used their median values to compartmentalise those variables and only included the records where their values were greater than the median (upper half of the median) [25,26]. This included the time the fire brigade spent to attend to the incident (i.e., 41 min), the percentage of property involved when the fire brigade personnel arrived (i.e., 1%), the number of fire personnel who attended incidents (i.e., 8 firefighters), the monetary value of damage loss (i.e., AUD 1000) and the length of stay in hospital (i.e., 1 day).

The hospital LOS was determined from the difference in days between the final discharge date and the date of admission in the index episode of care. Hospitalisations that consisted of multiple contiguous episodes of care for an injury event, where the discharge code was a transfer to another hospital or type change transfer, were considered as one hospital stay and were included in the total LOS calculation. 

### 2.2. Cohort Identification and Study Characteristics

The cohort for this study is residential fire-related incidents caused by smokers’ materials in the 10-year period between 2005 and 2014. It was determined using form of heat of ignition variable coded as cigarettes and Ignition factor variable entered as abandoned materials for the unclassified records of smokers’ materials category in the form of heat of ignition variable (*n* = 1361 records). In this study residential fire incidents in the cohort are referred to as smokers’ materials fire incidents. 

Residential fire-related records in health services and mortality data were determined by merging FRNSW AIRS with other data sources based on PPN and the date of the incident within the next 14 days of reporting the residential fire incident [27]. In this study, we reported the records based on incidents (unique incident IDs) for fire characteristics and health service utilisation, while death records were based on individual (unique PPN).

### 2.3. Statistical Analysis

Annual Australian adult smokers’ population estimates were used as an offset for the variables associated with individuals. An annual number of dwellings was used as an offset when we tested for the variables associated with incidents [28]. Since this study focuses on the impact of RFR cigarettes, fire incidents related to and caused by smokers’ materials are studied, and rates for death are calculated using the population of adult smokers in NSW [29]. The population of smokers aged 18 years and over in NSW were obtained from the ABS Australian Health Survey NSW. The number of smokers’ households was determined by using the average number of people in households (i.e., 2.6) for each year of the study and expressed as the number of cases per 100,000 population per year [30] [see Table S1]. 

This study was conducted based on an interrupted time-series design [31], which uses a categorical predictor, which in this study is RFR cigarettes regulation in 2010. Tested variables were plotted against time to inspect the data for outliers visually, and initial analysis of the data indicated that conditional variance exceeded the conditional mean value. This suggested that the data were over-dispersed and, therefore, negative binomial regression analysis of changes in residential fire-related incidents, corresponding deaths and health service utilisation and comparison of trends in pre- and post-RFR cigarettes regulation was conducted for count data. 

Analysis has been undertaken separately in pre-regulation and post-regulation of RFR cigarettes. Change of the relative rate of residential fire incident characteristics, deaths and health outcomes associated with the introduction of the RFR cigarettes mandate was estimated. Given that the rate of residential fire incidents had been decreasing before the RFR cigarettes mandates, the statistical question addressed is not just whether rates were lower after the placement of the regulation but whether the rate of decrease in residential fire incidents seems to be greater after the RFR cigarettes regulation. Incident Rate Ratio (IRR) was calculated with their 95% confidence intervals pre- and post-placement of the RFR cigarettes regulation. All analysis was conducted using R-3.6.0 (R Core Team, Vienna, Austria) [32] and STATA 16.0 (Stata Corp., College Station, TX, USA) [33].

## 3. Results

There were 43,707 residential fire-related incidents reported to FRNSW, of which 30% were caused by hot objects or friction, 17% by electrical fires, followed by fuelled fires (15%), open flames (12%) and smokers’ materials (4%). 

There were 2442 all-caused residential fire incidents reported to the fire brigade that required health service utilisation (involving one or more individuals during the 14-day time period after the incident), including 1939 ambulance use, 1497 ED visits, 795 hospital admissions and 86 burns clinic visits. There were 118 records of residential fire-related deaths from 2005 to 2014 (see Table 1 for details).

In the ten years to 2014, there were 1361 reported residential fire incidents caused by smokers’ materials in NSW of which 139 incidents required using health services. Health services included 99 records of ambulance use, 85 records of visiting ED, 68 records of admission to hospital and 5 records of a burns clinic visit due to residential fires caused by smokers’ materials. There were 13 records of deaths (see Table 1 for details). According to the study cohort, smokers’ materials were responsible for 3% of the residential fires and 11% of deaths.

Table 2 shows the result of testing the effectiveness of the RFR cigarettes regulation and changes in residential fire incidents based on different categories. Overall, there was an 8% reduction in (m) total residential fire incidents caused by smokers’ materials during the study period pre- and post-RFR cigarettes regulation (IRR = 0.92, 95% CI: 0.86–0.99). The number of fire incidents caused by abandoned smokers’ materials decreased by 8% post-regulation from 2010 to 2014; however, this change in numbers pre- and post-regulation was not significant [Table 2].

There was an 8% increase in the number of residential fires for which fire brigade personnel spent more than 41 min to attend from 2005 to 2010 and overall, 12% reduction comparing pre- and post-RFR regulation (IRR = 0.88, 95% CI: 0.79–0.98). There was a 13% reduction in the number of fire incidents that involved more than 1% of the property, when fire brigade personnel arrived (IRR = 0.87, 95% CI: 0.78–0.98).

Moreover, smokers’ materials fire incidents that damaged both contents and structure of the building decreased by 18% (IRR = 0.82, 95% CI: 0.71–0.93), and there was a 22% reduction in the number of fire incidents where fire flame extended beyond the room of fire origin (IRR = 0.78, 95% CI: 0.63–0.96). The changes in the number of residential fires with over AUD 1000 in monetary damage loss due to fire flames and firefighting reduced by 12% comparing pre- and post-regulation (IRR = 0.88, 95% CI: 0.78–0.99).

**Figure 2:**
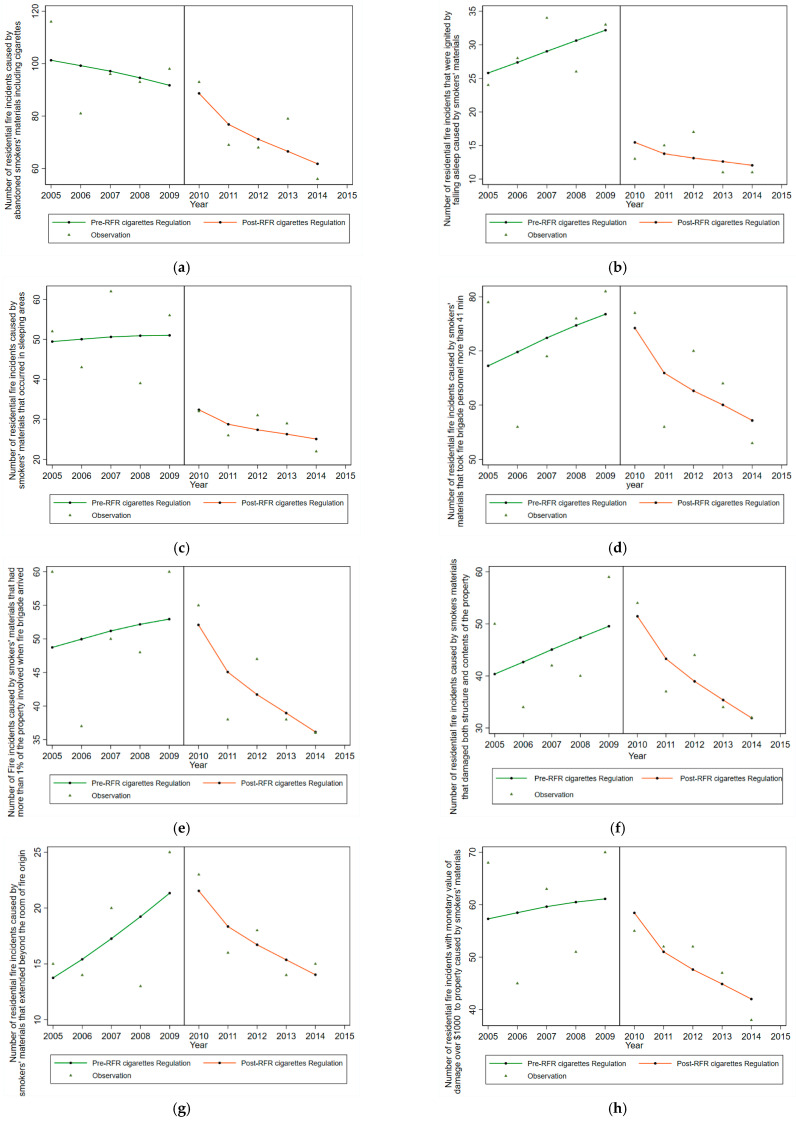

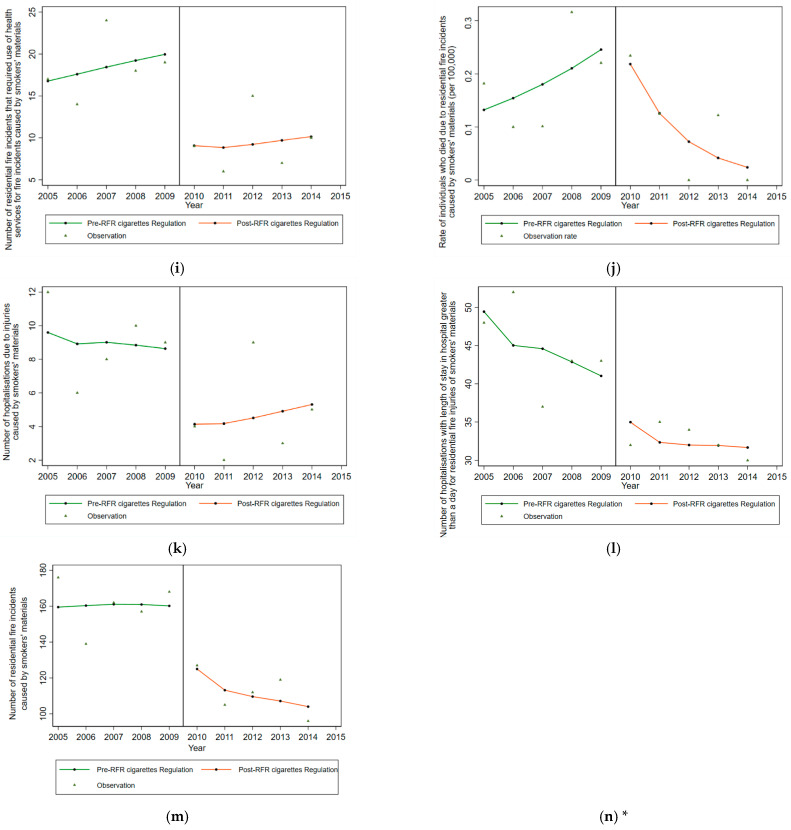
Estimated effect of RFR cigarettes regulation in the number of smokers’ materials fires using linked data, NSW, Australia, 2005–2014. (**a**) Number of residential fire incidents caused by smokers’ materials—abandoned cigarettes. (**b**) Number of residential fire incidents caused by smokers’ materials—ignited by falling asleep. (**c**) Number of residential fire incidents caused by smokers’ materials—in sleeping area. (**d**) Number of residential fire incidents caused by smokers’ materials—fire brigade spent more than 41 min. (**e**) Number of residential fire incidents caused by smokers’ materials—more than 1% of the property was involved in fire when brigade arrived (median percentage of property involved in fire). (**f**) Number of residential fire incidents caused by smokers’ materials—fire incidents that damaged structure and contents of the property. (**g**) Number of residential fire incidents caused by smokers’ materials—fire incidents that their flame damage extended beyond the room of fire origin. (**h**) Number of residential fire incidents caused by smokers’ materials—monetary value of damage loss greater than AUD 1000. (**i**) Number of residential fire incidents caused by smokers’ materials—health service utilisation. (**j**) Residential fire incidents caused by smokers’ materials—rate of individuals who died (per 100,000). (**k**) Number of residential fire incidents caused by smokers’ materials—hospitalisation. (**l**) Residential fire incidents caused by smokers’ materials—length of stay in hospital over a day (median LOS). (**m**) Residential fire incidents caused by smokers’ materials. (**n**) Residential fire incidents caused by smokers’ materials—number of brigade personnel attending fire incidents greater than 8 people. Note *: due to small numbers, no analysis could be performed.

Figure 2 illustrates results shown in Table 2 and each graph shows the observed rate (triangle) and the expected rate under the hypothesis of an effect of RFR cigarettes regulations (dots) estimated from a negative binomial model. The vertical line indicates the revision of RFR cigarettes regulations starting in 2010 [see Figure 2].

## 4. Discussion

Globally, several studies have indicated the positive impact of RFR cigarettes regulation on reducing cigarette-caused residential fires [9,11,34,35], and some studies concluded that implementation of fire-safe cigarettes is associated with reductions in residential fire mortality rates [10] and burns prevention [36]. On the other hand, some studies reported negative health impacts of RFR cigarettes. They indicate that RFR cigarettes may increase the presence of harmful and potentially harmful constituents (HPHCs) in mainstream smoke, mainly carbon monoxide, a few polycyclic aromatic hydrocarbons (PAHs) and tar [17,37,38]. The published toxicology studies to date have not reported any significant differences in toxicity between RFR or non-RFR cigarette papers. However, these studies have significant limitations and cannot be used to determine the potential toxicological differences between exposure to RFR or non-RFR cigarettes. Future research is needed to fully understand and evaluate the impact of RFR cigarettes on smokers’ health and life. 

There are also studies such as Bonander et al.’s that did not find RFR cigarettes effective to reduce health adverse outcomes [18]. In that study, they found no significant effects on all-cause fire mortality, residential fire mortality and cigarette-caused fire mortality along 50 US states. They listed some limitations in their study in terms of the data source (i.e., National Fire Incidents Data Reposting System—NFIRS) they used, which includes having an incomplete coverage within each state, with a high proportion of missing values on what caused fires. Similarly, we found no significant changes in death rate pre- and post-regulation, and number of health service utilisation, hospitalisation and length of stay in hospital did not differ significantly either. 

To our knowledge, this is the first population-based study that investigated the impact of RFR cigarette regulation in terms of residential fire-related incidents and health outcomes using linked data. In this study, we observed a significant reduction in the number of residential fire incidents ignited by abandoned cigarettes post-RFR cigarettes regulation that is attributed to human behaviour. Therefore, changes in human behaviour such as not discarding lit cigarette butts as well as RFR cigarettes that self-distinguish could play a part in the reduction of such incidents. 

There were no significant changes in the number of fire incidents ignited by falling asleep or in sleeping areas. These are both attributed to human behaviour, especially smoking while in bed causing deep burns, inhalation injury and poisoning with combustion products [39]. RFR cigarettes are self-extinguished before they burn to completion but cigarettes can start a fire before it burns its full length depending on the type of substance they are in contact with. Studies have shown that modern furnishing results in faster fire development.

The severity of fire incidents was tested using the number of personnel, time spent to attend fire incidents, percentage of property involved in the fire when fire brigade personnel arrived, monetary loss due to fire damage and firefighting, type of incident damaging both structure and contents and the extent of flame damage beyond the room of fire origin. It was shown that there was a reduction observed in the number of fire incidents in those variables post-RFR cigarettes regulation. 

The number of residential fire incidents that extended beyond the room of fire origin and time fire brigade personnel spent to attend fire incidents increased significantly pre-RFR cigarettes regulation. One of the possible reasons might be the materials that are used in modern buildings and furnishing, which accelerate the speed of fire damage in the properties [40,41]. It is expected that the building furnishings are the cause of fire extending beyond the room of origin, as well as increasing firefighting time as shown in a previous study investigating cigarette fires involving upholstered furniture in residences [42]. Our results align with previous studies in terms of smokers’ materials and specifically cigarette fires impact and clearly shows the positive impact of RFR cigarettes regulation. 

According to our study cohort, the rate of health service utilisation and deaths did not change post-regulation. These results emphasize the importance of other fire intervention strategies alongside RFR cigarettes to reduce the negative health impact of residential fire incidents caused by smokers’ materials. 

Even though the health outcome of residential fire incidents caused by smokers’ materials did not reduce significantly over the study period after the RFR cigarettes regulation placement, the number of fire incidents caused by smokers’ materials and the intensity of those fires have reduced. This indicates that the fire safety regulation and the standards have worked well and positively impacted the residential fire incident outcomes. 

It is important to note that a crucial aspect of fire safety measures’ regulation is monitoring the performance and regulatory compliance. Following the coronial investigation of a fatal residential fire incident in 2015 in NSW, even though the cause of the fire remained unknown, expert evidence established that the cause of the fire incident was an unextinguished cigarette butt of one of the tenants in the apartment blown into a waste bin [43], which probably had been smuggled to Australia. 

### Limitations

This study identified some limitations. Fire incidents caused by cigarettes and abandoned smokers’ materials were identified using FRNSW AIRS data; therefore, the cause of residential fire-related records that were identified in other data sources was unknown and not included in our analysis. In addition, unreported residential fires were not included in this study [27,44], as well as those who treated their residential fire-related injuries by accessing medical centres, general practitioners and pharmacies. The quality of linked data and administrative health data are other limitations of this study [23,45]. 

Another limitation of this study is using slightly dated data sources. FRNSW AIRS data contains records of fire incidents that were attended by Fire and Rescue NSW, and there was a change in the platform for coding of the reporting, and for NSW, the latest figures available were from 31 March 2015, and for the study, we used a 10-year period from 2005 to 2014 for consistency in reporting the data. The process of linking the different datasets and obtaining appropriate data custodians’ approvals also results in significant time delay. Additionally, more broadly, in NSW and Australia, there have not been any major regulatory changes since 2015 that would affect the building standards or the appliances/furniture, rendering the data in this study still relevant. Future studies may explore the impact of RFR cigarettes regulation and other safety measures with more recent linked data sources.

It is important to note that having wide intervals for the “death” variable is because of the small number of deaths records, which increases the degree of uncertainty. The wide ranges of intervals indicate that we have little knowledge about the effect and that further information is required. 

## 5. Conclusions

The impact of RFR cigarettes regulation, placed in 2010, was examined using FRNSW data linked to the health administrative dataset from 2005 to 2014. This study adds to the body of evidence that demonstrates RFR cigarettes regulation has a positive impact on the residential fire incidents outcome. Unlike previous studies that have used fire brigade and/or mortality data to examine the impact of RFR cigarettes regulation, this study utilised linked data, which provided a comprehensive picture of each fire incident in terms of the type of incident, type of fire and its impact on the property and associated health impact on individuals. This provides evidence of the government’s intervention in fire risk protective measures and bestows some direction for other fire safety policies and regulations.

This study measured RFR cigarettes regulation impact for five years after placement of the regulation; however, there may be many other factors contributing to the result of changes in the number of severe cigarette-related fires such as fewer smokers and improvement in fire safety measures. Future studies may explore a more recent data to test if the reduction in severity of fire incidents has been consistent over the years. 

## Figures and Tables

**Table 1:** Number of residential fire incidents and associated health utilisation services and deaths using FRNSW AIRS linked to health administrative dataset, NSW, Australia, 2005–2014.

Year	Residential Fire incidents	Hot Object or Friction |	Electrical Equipment |	Fuel- Fired |	Open Flame |	Smokers’ Materials (including Cigarettes, Cigars, Pipes, etc.) |	Cigarettes Fires ||	Undetermined ||	Abandoned Undetermined Smokers’ Materials |||	Study Cohort (Smokers’ Materials)	Health Service Utilisation (Total)	Health Service Utilisation (Cohort)	Deaths (Total)	Death (Cohort)
2005	4424	1333	690	585	567	201	159	41	17	176	217	17	16	*
2006	4465	1386	674	639	581	165	128	36	11	139	267	14	12	*
2007	4748	1431	802	653	510	177	142	34	20	162	292	24	11	*
2008	4497	1372	772	654	518	180	149	31	8	157	266	18	17	*
2009	4553	1367	761	668	513	196	157	38	11	168	292	19	13	*
2010	4410	1403	805	647	442	146	96	31	16	127	238	9	10	*
2011	4233	1165	827	673	483	122	45	39	22	105	240	6	7	*
2012	4340	1255	715	646	541	132	31	38	19	112	254	15	13	*
2013	4118	1163	724	617	463	141	38	40	18	119	194	7	12	*
2014	3919	1066	792	594	451	117	43	34	14	96	182	10	7	*
Total	43,707	12,941	7562	6376	5069	1577	988	362	156	1361	2442	139	118	13

* Records are <5. We are not allowed to report records that were less than five for ethical reasons to avoid the risk of re-identification. | Form of heat of ignition variable categories. || Smokers’ materials subcategories. ||| Smoker’s materials records that were undetermined in form of heat of ignition variable and were considered abandoned materials according to Ignition factor variable.

**Table 2:** Estimated effect of RFR cigarettes regulations in the number of smokers’ materials fires using linked data, NSW, Australia, 2005–2014.

Residential Fire Incidents Caused by Smokers’ Materials	RFR Cigarettes’ Pre-Regulation IRR (95% CI)	RFR Cigarettes’ Post-Regulation IRR (95% CI)	Ratio of Slopes IRR (95% CI), *p* Value
(a) Number of residential fires due to abandoned cigarettes	1.01 (0.95–1.09)	**0.92 (0.86–0.99)**	0.91 (0.82–1.00), *p* = 0.05
(b) Ignited by falling asleep for residential fires caused by smokers’ materials	1.10 (0.98–1.23)	0.95 (0.80–1.12)	0.86 (0.70–1.06), *p* = 0.15
(c) Number of residential fires in sleeping areas caused by smokers’ materials	1.05 (0.95–1.16)	0.95 (0.84–1.06)	0.90 (0.78–1.04), *p* = 0.17
(d) Number of residential fires for which fire brigade spent more than 41 min	**1.08 (1.00–1.16)**	0.94 (0.87–1.02)	**0.88 (0.79–0.98), *p* = 0.02**
(e) Number of residential fires that had more than 1% of the property involved when fire brigade arrived	1.06 (0.96–1.18)	0.92 (0.84–1.01)	**0.87 (0.76– 0.98), *p* = 0.03**
(f) Number of residential fire incidents that damaged structure and contents of the property	1.09 (0.98–1.22)	**0.89 (0.81–0.99)**	**0.82 (0.71–0.93), *p* < 0.01**
(g) Number of residential fire incidents with flame damage beyond the room of fire origin	**1.16 (1.00–1.35)**	0.91 (0.78–1.05)	**0.78 (0.63–0.96), *p* = 0.02**
(h) Number of residential fire incidents with monetary value of damage over AUD 1000 due to fire and firefighting	1.06 (0.96–1.17)	0.93 (0.85–1.01)	**0.88 (0.78–0.99), *p* = 0.03**
(i) Health service utilisation	1.09 (0.94–1.26)	1.04 (0.84–1.27)	0.95 (0.74–1.22), *p* = 0.71
(j) Deaths	1.17 (0.73–1.86)	0.58 (0.25–1.32)	*
(k) Hospitalisations	1.02 (0.83–1.26)	1.07 (0.78–1.48)	1.05 (0.74–1.49), *p* = 0.79
(l) Length of stay in hospital	1.00 (0.91–1.10)	0.98 (0.88–1.10)	0.98 (0.85–1.13), *p* = 0.80
(m) Total number of residential fire incidents caused by smokers’ materials	1.04 (0.99–1.09)	0.96 (0.91–1.02)	**0.92 (0.86–0.99), *p* = 0.04**
(n) Number of residential fire incidents that required more than 8 fire brigade personnel to attend them	*	*	*

* No analysis could be performed due to small numbers. Bold values denote statistical significance at the *p* < 0.05 level.

## Data Availability

The data presented in this study are not publicly available due to sensitive health and personal data and medical confidentiality.

## Supplementary Materials

The following supporting information can be downloaded at: https://www.mdpi.com/article/10.3390/ijerph191912481/s1, Table S1: Total population of NSW, total number of dwellings in NSW, total number of dwellings that are FRNSW responsibility, total population of adult smokers in NSW, total number of dwellings for adult smokers in NSW and total number of dwellings.
