# Supplementary material for: The Impact of Reduced Fire Risk Cigarettes Regulation on Residential Fire Incidents, Mortality and Health Service Utilisation in New South Wales, Australia
Source: Int J Environ Res Public Health. 2022 Sep 30;19(19):12481. doi: 10.3390/ijerph191912481 (PMC9566670; doi:10.3390/ijerph191912481)
Supplement: Supplementary file 1 [file ijerph-19-12481-s001.zip › ijerph-1890038-supplementary.pdf]

Supplementary Materials:

Table S1. Total population of NSW, total number of dwellings in NSW, total number of dwellings that are FRNSW responsibility, total population of adult smokers in NSW, total number of dwellings for adult smokers in NSW and total number of dwellings.

Year	NSW Population	NSW Dwellings	Dwellings within FRNSW responsibility	Smokers percentage in population	Smokers population	Smokers dwellings
2005	5104524	2,699,608	2,429,647	20.8	1061741	408361.9
2006	5150878	2,728,719	2,455,847	19.9	1025025	394240.3
2007	5231887	2,755,881	2,480,293	18.9	988826.6	380317.9
2008	5331249	2,783,314	2,504,983	17.8	948962.3	364985.5
2009	5430489	2,811,020	2,529,918	16.7	906891.7	348804.5
2010	5509085	2,839,002	2,555,102	15.5	853908.2	328426.2
2011	5577052	2,864,531	2,578,078	14.4	803095.5	308882.9
2012	5648682	2,903,545	2,613,190	14.3	807761.5	310677.5
2013	5733451	2,943,090	2,648,781	14.3	819883.5	315339.8
2014	5821687	2,983,173	2,684,856	14.2	826679.6	317953.7
